# Improved Tracking and Resolution of Bacteria in Holographic Microscopy Using Dye and Fluorescent Protein Labeling

**DOI:** 10.3389/fchem.2016.00017

**Published:** 2016-04-19

**Authors:** Jay L. Nadeau, Yong Bin Cho, Jonas Kühn, Kurt Liewer

**Affiliations:** ^1^Graduate Aerospace Laboratories, California Institute of TechnologyPasadena, CA, USA; ^2^Department of Biomedical Engineering, McGill UniversityMontreal, QC, Canada; ^3^Institute for Astronomy, ETH ZürichZürich, Switzerland; ^4^Jet Propulsion Laboratory, California Institute of TechnologyPasadena, CA, USA

**Keywords:** digital holographic microscopy, interferometric microscopy, quantitative phase imaging, bacterial tracking, bacterial motility, green fluorescent proteins, Mach-Zehnder

## Abstract

Digital holographic microscopy (DHM) is an emerging imaging technique that permits instantaneous capture of a relatively large sample volume. However, large volumes usually come at the expense of lower spatial resolution, and the technique has rarely been used with prokaryotic cells due to their small size and low contrast. In this paper we demonstrate the use of a Mach-Zehnder dual-beam instrument for imaging of labeled and unlabeled bacteria and microalgae. Spatial resolution of 0.3 μm is achieved, providing a sampling of several pixels across a typical prokaryotic cell. Both cellular motility and morphology are readily recorded. The use of dyes provides both amplitude and phase contrast improvement and is of use to identify cells in dense samples.

## Introduction and background

Digital holographic microscopy (DHM) is an emerging technique of relevance to fields of biology, chemistry, and physics where instantaneous sampling of a three-dimensional volume is desired. Rather than focusing at a single sample plane, as in light microscopy, DHM encodes the amplitude and phase of the entire sample volume as a hologram, or interference pattern, resulting from the perturbation of the probe light beam by the sample.

The optical components involved in DHM are relatively simple and low-cost compared to those needed for fluorescence microscopy. Because nearly all of the illuminating photons are captured, low-power excitation sources can be used—specifically milliwatt diode lasers, which are available in a large range of wavelengths. For similar reasons, camera sensitivity can be significantly less than for fluorescence imaging; cooled CCDs are not required. For capture of rapid motion, cameras with full-frame shutter are preferable to rolling readout. Spatial resolution is determined by the numerical aperture of the system, with a limiting resolution of λ/NA as in conventional imaging as long as the camera pixels sample the fringes at the Nyquist frequency or better. Because a single wavelength of illumination light is used, compound objective lenses with chromatic aberration correction are not needed. Extremely low cost instruments may be constructed using web cameras, light-emitting diode illumination, and simple aspheric lenses (Lu et al., 2014; Mico et al., 2014). While DHM is usually used in transmission mode for imaging through low-density samples, it is possible to build instruments with similar components to operate in reflectance mode (Colomb et al., 2010; Lee et al., 2011; Yaqoob et al., 2011).

After holograms are recorded, reconstruction of quantitative phase, and amplitude images may be numerically performed at selected z-planes throughout the volume or at all z-planes (Ferraro et al., 2003; Dubois et al., 2004; De Nicola et al., 2005; Marquet et al., 2005). Amplitude images are equivalent to transmission light microscopy; quantitative phase images have no direct counterpart in light microscopy and are related to the product of the cell thickness *h* and the difference in refractive index between the medium (*n*_*m*_) and cell (*n*_*c*_) (Rappaz et al., 2005):

(1) $$\begin{array}{l} △\varphi =\frac{2\pi }{\lambda }h\left(x,y\right)\left[n_{c}\left(x,y\right)-n_{m}\right] \end{array}$$

where λ is the wavelength of illumination.

The difference in refractive index between cell cytoplasm and ordinary aqueous cell culture medium is very small. However, changes in the refractive index can occur as cells take up or lose water, thus generating signals under phase imaging. Processes that can be observed this way include cell cycle arrest, initiation of apoptosis, excitotoxicity, and neuronal response to glutamate stimulation (Dubois et al., 2006; Kemper et al., 2006; Jourdain et al., 2011; Pavillon et al., 2012; Falck Miniotis et al., 2014; Rappaz et al., 2014).

All of these studies have been performed in large, eukaryotic cells with distinct membrane-bound organelles. Only a few DHM studies of prokaryotes have been reported, largely because of the difficulty of identifying the cells under either amplitude or phase due to their small size and low contrast (both *h* and Δ*n* in Equation 1 are small). For applications for which optimized contrast is more important than phase retrieval (e.g., bacterial tracking), phase contrast may be enhanced by immersion of the sample into a high-index medium such as glycerol or by labeling cells with dyes. Addition of a dye causes a change in the cell's absorbance spectrum, which is related to the imaginary part of the refractive index, *n*_*img*_. The change in the real part *n*_*real*_ can then be calculated using the Kramers–Kronig relation (Cherkezyan et al., 2012; Gaigalas et al., 2013):

$$\begin{array}{l} n_{real}\left(\lambda \right)=\frac{-2\lambda^{2}}{\pi }P\int_{\delta }^{\infty }\frac{n_{img}\left(\lambda^{{}^{\prime }}\right)}{\lambda^{{}^{\prime }}\left(\lambda^{{}^{\prime }2}-\lambda^{2}\right)}d\lambda \left(2\right), \end{array}$$

where P represents the Cauchy principal value and the value δ is chosen to avoid divergence at λ = 0. Since the refractive index change is an integral over the absorption, the greatest change in *n* will be seen at wavelengths larger than the principal absorbance peak of the dye. Thus, an illumination wavelength should be chosen that is redder than the dye's absorbance peak. We have recently shown that the metallo-corrole dye Ga(tpfc)(SO_3_H)_2_, which has a strong absorbance peak at 400 nm, produces significant phase signal enhancement with 488 nm illumination of labeled bacterial cells (Nadeau et al., 2015). However, this was the only dye investigated, and attempts to increase amplitude contrast with dyes were not done in that initial study.

Different considerations apply for amplitude imaging. Most biological cells are phase objects and thus nearly transparent in amplitude. The exception is when the probe wavelength is highly absorbed by the cells due to the presence of a pigment, in which case the amplitude image may show greater contrast. For example, chlorophyll has an extinction coefficient of >10^5^ mol^−1^ cm^−1^ at 405 nm, so photosynthetic cells imaged with a violet laser appear dark (Kühn et al., 2014). Labeling cells with a dye that absorbs at the illumination wavelength should theoretically produce the same effect, but this has not yet been explored for DHM.

When imaging dense population, the signal-to-noise in holographic images is dominated by sample chamber depth and organism concentration, being inversely linearly proportional to both (Meng et al., 1993; Meng and Hussain, 1995; Pu and Meng, 2004). For our previously reported instrument, this corresponded to a limiting concentration of ~10^8^ cells/mL for *Escherichia coli*. At this concentration, the signal to noise value was ~1 (Kühn et al., 2014). Maximizing the resolvable concentration allows for investigation of cell-cell interactions and for the most efficient collection of trajectory data.

In this paper, we explore the ability of several fluorescent probes to increase amplitude and phase contrast in high resolution DHM on both thin slides and in thicker, crowded chambers. The instrument is a custom Mach-Zehnder off-axis DHM specifically adapted for maximum spatial resolution by the choice of laser illumination wavelengths (405 and 488 nm) and high-index, long-working-distance objective lenses. It is described for the first time in this paper. We also explore several dyes not previously tested with DHM. NanoOrange is a protein quantitation tool that has been shown to label bacterial flagella in fluorescence microscopy. With an absorption peak at 460 nm, this dye changes phase contrast only slightly, but markedly increases amplitude contrast of cell boundaries, although not to the point where flagella can be resolved. The porphyrin dye zinc tetraphenylporphyrin (ZnTPP) is shown to increase both amplitude and phase contrast, particularly in cell membranes. The cyan fluorescent protein Cerulean, on the other hand, appears to have almost no effect on either amplitude or phase, although it is shown to marginally increase detectability of cells in dense samples.

Several different strains are used in these studies in order to demonstrate the generality of the approach as well as to highlight the diverse cellular and subcellular structure of micrometer-sized organisms. *Vibrio alginolyticus* is a very small, highly motile marine bacterium with a single polar flagellum. *Bacillus subtilis* is a larger prokaryote, with cells 5 μm long, and is peritrichous, showing multiple flagella. *E. coli* is used to express fluorescent protein because of the ease of transfection of this organism; it is also small and peritrichous. Finally, we look at *Picocystis salinarum*, a picoplanktonic green alga that represents the lower limit of eukaryotic cell size (~2 μm diameter) (Lewin et al., 2000). Its cell is dominated by chloroplasts and so allows evaluation of the amplitude and phase contrast provided by chlorophyll. It is a coccoid alga and non-motile.

## Materials and methods

### Instrument description

**Figure 1:**
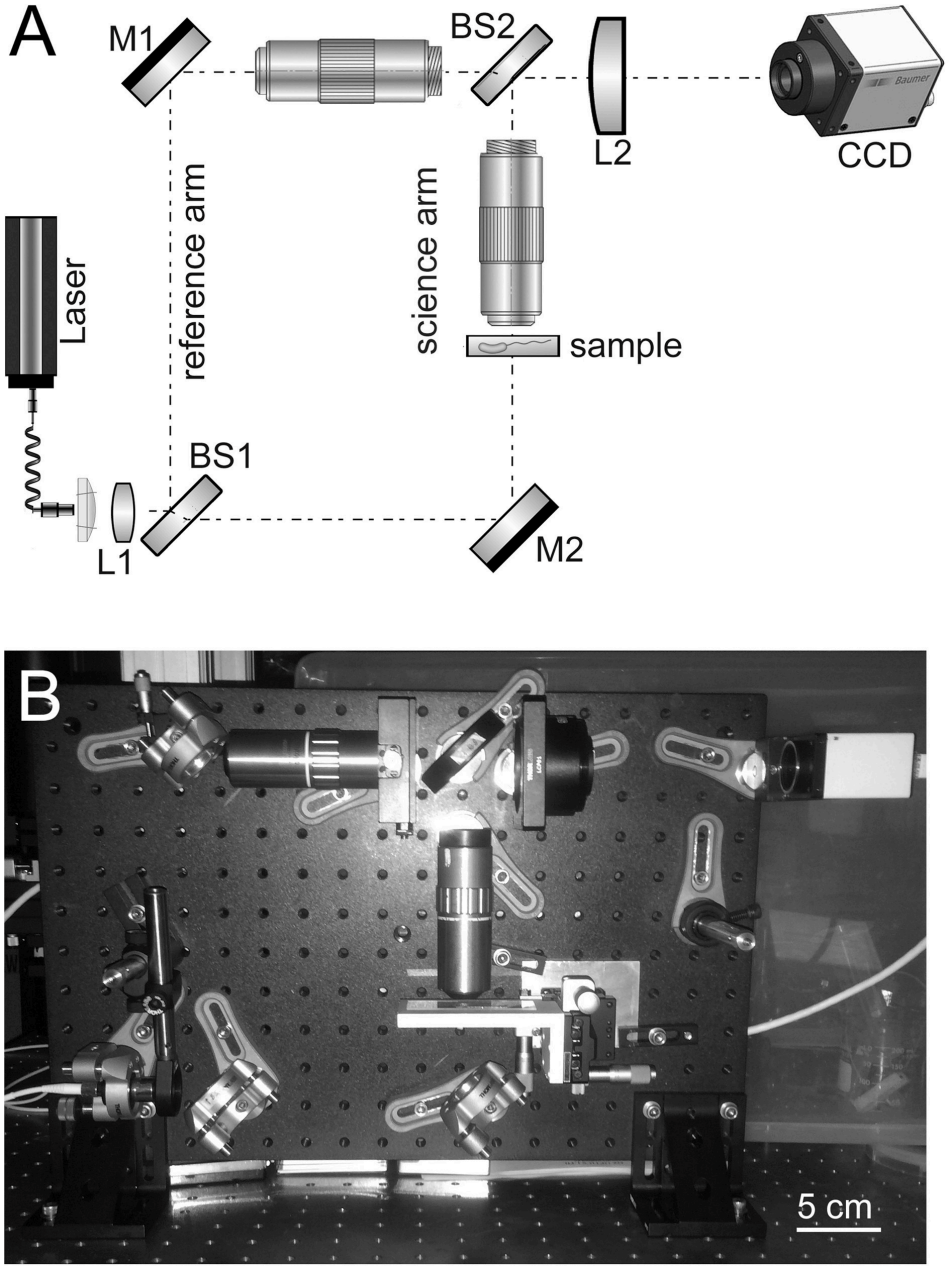
**(A)** Schematic of high-resolution instrument developed and used in this owrk. M, mirror; L, lens; BS, beamsplitter. **(B)** Photo of assembled instrument including camera.

The design of the high-resolution microscope is based on a modified Mach–Zehnder configuration. Figure 1 shows a schematic and photograph of the instrument. This design was chosen since the science path and the reference path are well separated and this provides a large easily accessible sample area and accommodates the large microscope objectives that were used. The microscope objectives used are Mitutoyo 100x HR objectives with a numerical aperture (NA) of 0.7. The objective is infinity corrected so an achromatic field lens of 200 mm focal length is used to form the image on the CCD. The second microscope objective is inserted in the reference arm and serves two purposes. It provides a reference beam with the same curvature as the science beam and, since it has an identical lens, it provides an equal thickness of glass. It is important for the reference beam and the science beam to have about the same optical path length (OPL) since the coherence length of the diode laser being used is relatively short and unstable in time.

With the retained 200 mm field lens the effective magnification of the system is 78, which provides ~44 nm per 3.45-μ m pixel sampling on the CCD. The lateral resolution of the instrument was not directly measured due to lack of a suitable target, but it easily resolves the smallest 780 nm lines of the group 9 element 3 of a high-resolution USAF resolution test target. The diffraction-limited resolution is estimated to be ~0.3 μm at 488 nm with 0.7 NA.

The illumination originates from a single-mode fiber coupled diode laser that is collimated before the first beamsplitter. Both 405 and 488 nm wavelengths were used. A 400 mm focal length singlet (L1) was added just after the collimator to increase the laser intensity and avoid vignetting on the front aperture of the objectives.

The fold mirror in the reference arm (M1) just before the objective is on a small translation stage that can be moved to adjust OPL in the reference arm. The first beamsplitter (BS1) and the mirror in the reference arm are adjustable in angle so that shear and pointing in that arm can be adjusted. Once set, these rarely need to be adjusted unless large changes are made to the OPL.

The second beamsplitter (BS2) recombines the beams before they impede on the CCD. The second beamsplitter's angle is adjusted to give a fringe spacing of around 4 pixels/fringe on the CCD.

### Bacterial strains

*V. alginolyticus* (gift of R. Stocker, MIT) was maintained by serial passage into 2216 Marine Broth (Difco) and cultured at 30°C with agitation at 200 rpm. *B. subtilis* ATCC6051 was purchased from the American Type Culture Collection (Manassas, VA) and maintained in lysogeny broth (LB) (Difco). Two strains of *E. coli* were used: a control strain (AW405) and a cloning strain (Top-10) transfected with a plasmid encoding the Cerulean fluorescent protein under the control of an ampicillin-promoter (gift of T. Talisman, City of Hope). Both strains were maintained at 37°C in LB, with 100 μg/mL ampicillin added to the cerulean strain. Optical density measurements were taken of each culture to ensure that similar densities were used for imaging, since the cells subjected to ampicillin selection grew more slowly. *P. salinarum* was collected from Mono Lake, California and maintained in its native water at ambient temperature for < 24 h before imaging.

For labeling, mid-log cultures were washed by gently sedimenting at ~1000 × *g* in a microcentrifuge and resuspending in marine motility medium (for *V. alginolyticus*: Tris buffer [50 mM Tris-HCl, pH 7.5], 300 mM NaCl, 5 mM MgCl2, 5 mM glucose; for other strains, 10 mM potassium phosphate, 0.1 mM EDTA, 10 mM NaCl, pH 7.5). For DHM, cells were diluted in motility medium to an optical density of ~0.05, corresponding to an ~20-fold dilution of a mid-log culture or about 5 × 10^7^ cells/mL. The diluted culture was sandwiched between two optical glass slides or coverslips with or without a 0.6 mm deep reservoir provided by a silicone gasket. Mono Lake samples were unprocessed. Imaging was performed at room temperature.

### Dyes and fluorescent proteins

Dye spectra were recorded on a SpectraMax plate reader (Molecular Devices) and fluorescently labeled bacteria were visualized on an Olympus IX-60 inverted epifluorescence microscope using a Quantum Dot filter set (emission: 420/80 nm, dichroic 475 nm, emission 500/longpass) and a 100x 1.4 NA oil immersion objective. Cerulean-expressing *E. coli* and *P. salinarum* were visualized on a Zeiss 510 confocal (Beckman Imaging Center) with a 63x 1.4 NA oil immersion objective and excitation with the 488 nm line of an Ar ion laser.

For labeling with NanoOrange, 10 μL of a NanoOrange stock solution in DMSO (Molecular Probes) was added to washed cells diluted 100-fold into motility medium. The cells were rocked for 20 min in the dark, then washed by centrifugation and resuspension in motility medium, and mounted onto slides using ProLong Gold mounting medium (Molecular Probes) for epifluorescence microscopy or used for DHM immediately. Labeling with zinc tetraphenylporphyrin (ZnTPP, Sigma) was using a 30 mM stock solution added as 100x for a final concentration of 30 μM for 1 h. The cells were then washed twice by pelleting and resuspension in motility medium before imaging.

### DHM data acquisition and analysis

Real-time hologram acquisition at 4 M pixel resolution, and a-posteriori intensity and phase numerical reconstructions for various focus distances, were performed using the Koala software (LynceeTec). Additional post-processing of reconstructed images was performed with the software Fiji (Schindelin et al., 2012).

## Results

### Appearance of dyed strains under epifluorescence

**Figure 2:**
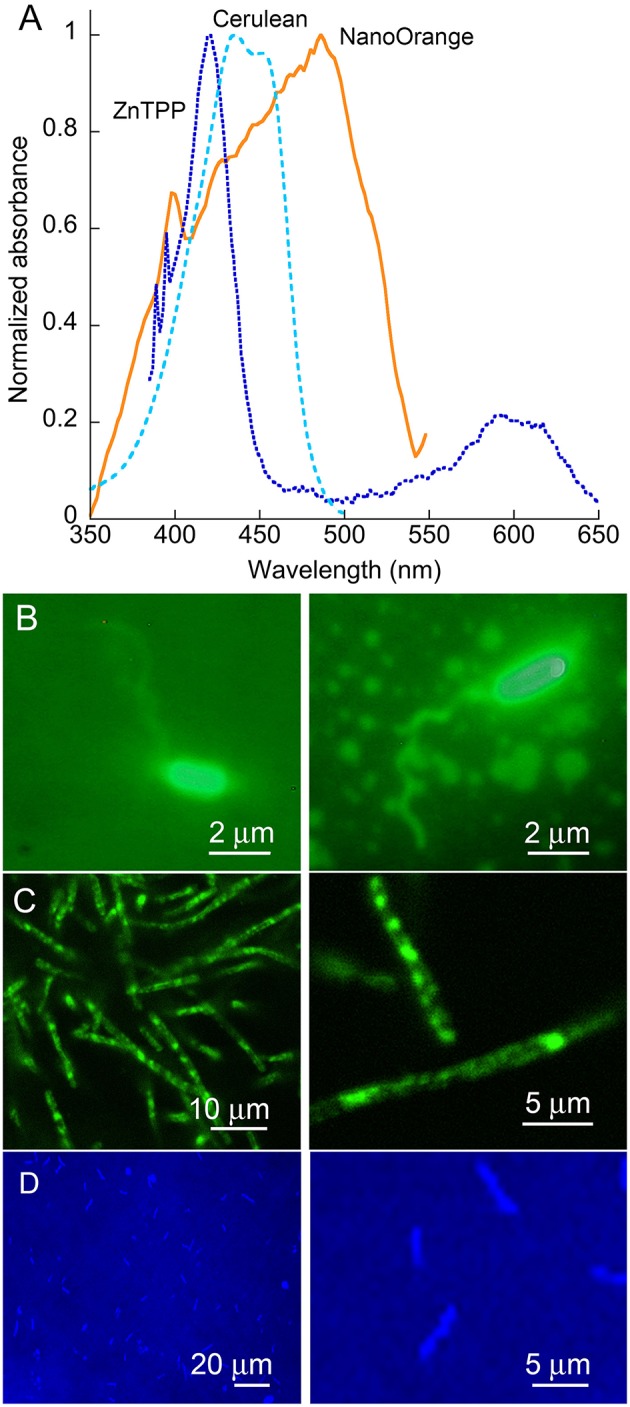
**Dyes and organisms used. (A)** Absorbance spectra of the two dyes and one fluorescent protein used in this work. Note the strong Soret band of the porphyrin ZnTPP, and the broad absorbance of NanoOrange. **(B)** Epifluorescence images of *V. alginolyticus* labeled with NanoOrange. **(C)** Epifluorescence images of *B. subtilis* labeled with NanoOrange. Labeling patterns with ZnTPP were similar (not shown). **(D)** *E. coli* expressing the Cerulean fluorescent protein.

Absorption and emission spectra of the probes used are given in Figure 2A. It can be seen that while the wavelength we used for illumination, 488 nm, is off the absorbance peak of NanoOrange, it still falls in a highly absorbing region of the absorbance spectrum. For ZnTPP, the 488 nm illumination wavelength falls to the red side of the strongest absorbance band. For Cerulean, illumination was performed at 405 nm for optimum absorbance. All of these dyes' fluorescence is emitted in the green (Cerulean), orange (NanoOrange), or red (ZnTPP) and does not alter DHM imaging, as the fluorescence signal is both temporally incoherent and comparatively very weak relative to the excitation band, though it can serve as a useful confirmation of successful labeling using fluorescence microscopy in parallel. With *V. alginolyticus*, cell bodies were strongly labeled with NanoOrange, and sufficient dye was bound to flagella to enable their visualization under ordinary widefield epifluorescence microscopy (Figure 2B). With *B. subtilis*, flagella were not seen, but substantial internal structure could be visualized using either NanoOrange or ZnTPP (Figure 2C)*. E. coli* expressing Cerulean showed a uniform pattern of expression across the culture (Figure 2D).

### DHM in amplitude and phase

#### Vibrio alginolyticus

This was the smallest of the test organisms (1 × 2 μm), and subcellular structures, including flagella, were not resolved in either dyed or undyed cells. However, the cells were clearly resolved in both amplitude and phase. Dye labeling did not qualitatively change the cells' appearance, but did significantly increase contrast.

**Figure 3:**
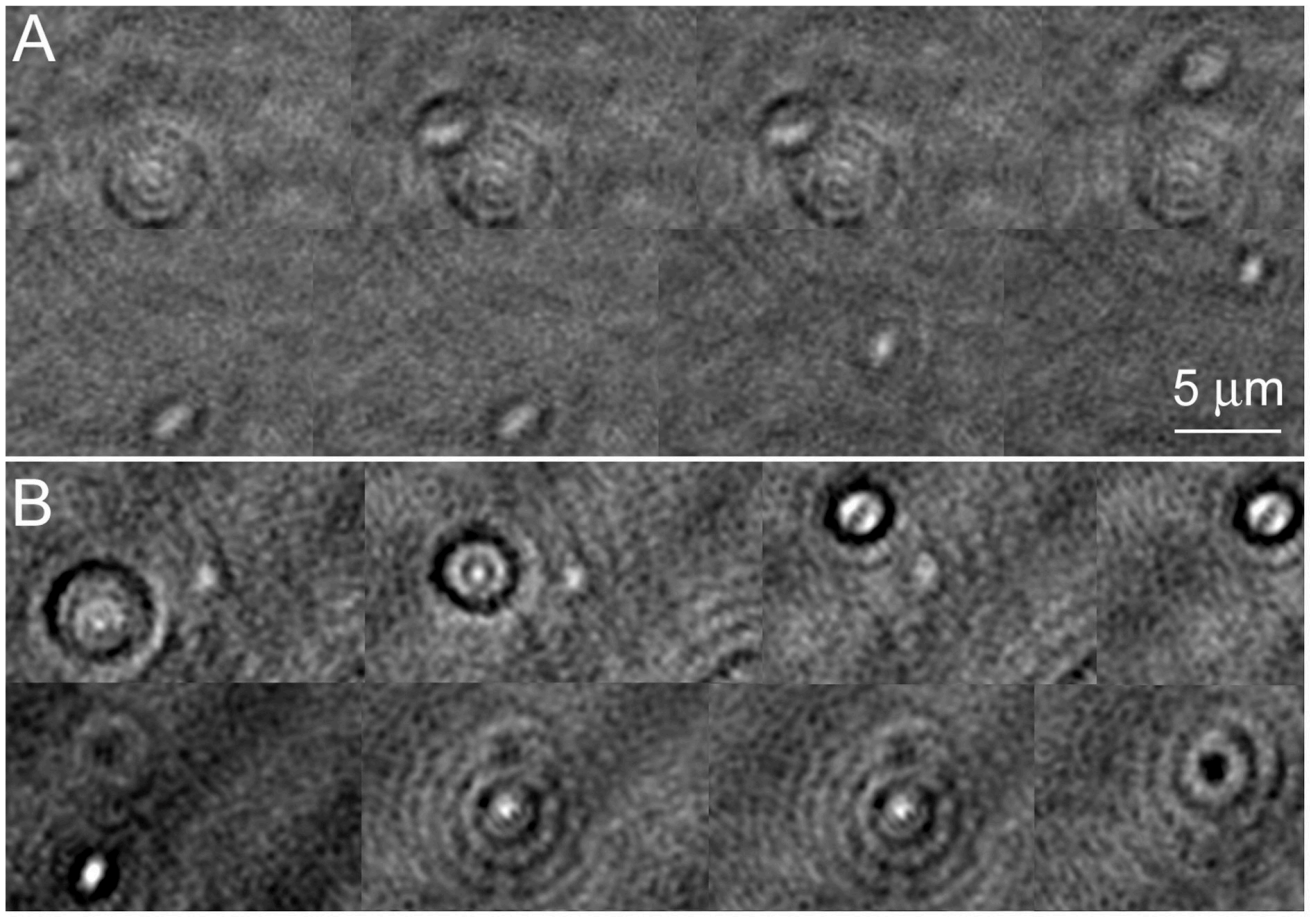
**Amplitude images of ***Vibrio alginolyticus*** swimming through x, y, and z**. Images show a time series in a single z-plane with a cell coming into/going out of focus as it swims. The images are successive frames recorded at 7 frames per second, or 0.14 s between frames. **(A)** No dye. **(B)** Labeled with NanoOrange.

Amplitude reconstructions of the holograms showed cells traveling through different z-planes as they swam. They moved in and out of focus very rapidly. Figure 3A shows an amplitude image of a *V. alginolyticus* cell as it moved. Picking the best focus was not usually apparent by eye. When in focus, the individual bacterial cells appeared nearly featureless. In the upper part of the panel, cells slightly out of focus may be seen; the lower part of the panel shows the best focus (also see Video 1). The effects of labeling with NanoOrange are shown in the lower half of the figure. Figure 3B shows a labeled cell traveling through z. It can be appreciated that the diffraction pattern of the cell was significantly more pronounced than in the unlabeled case, even when the cell was substantially out of focus. At “best” focus, the cell appeared featureless, but darkly outlined. The cell contained a central feature that switched from bright to dark as the focus changed. The dyed cell remained visible through a larger depth of focus than the unlabeled cell (also see Video 2): where the unlabeled cell was visible through ~10 focal planes in z (~3.5 μm) before its signal to noise became 1, the dyed cell was clearly visible throughout the sampled z volume (~10 μm).

**Figure 4:**
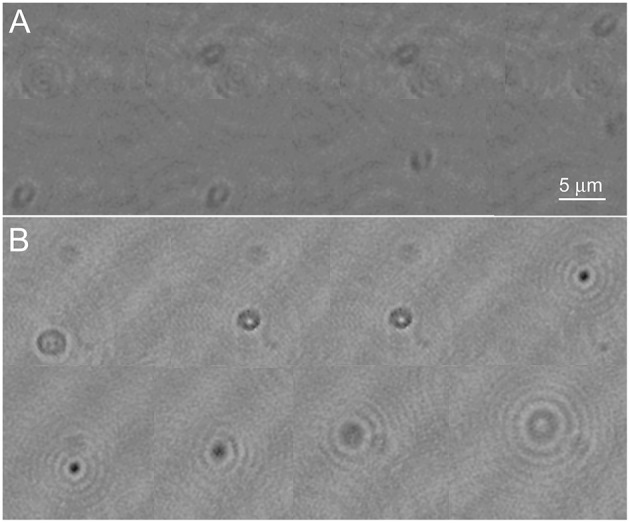
**Phase images of ***Vibrio alginolyticus*** swimming through x, y, and z. (A)** No dye. **(B)** Labeled with NanoOrange. Displayed phase range is −pi to +pi. As in Figure 3, the images are successive frames with 0.14 s between each frame.

In the phase images, the most obvious effect of the labeling was that the cell remained detectable through a larger z-depth than an undyed cell (Figure 4A shows an unlabeled cell and Figure 4B a NanoOrange-labeled cell; also see Videos 3, 4). The central focus point shifted from bright to dark (positive/negative phase offset) as the cell passed through focus. This is a manifestation of the so-called Gouy phase anomaly (Gouy, 1890; Feng and Winful, 2001) as an object passes through the geometric focus. It has been previously described in detail in the context of DHM (Wilson and Zhang, 2012), and used for precise z-localization of weakly scattering objects for tracking (Wilson et al., 2013). Labeling with ZnTPP produced qualitatively similar results to what was seen with NanoOrange (not shown).

Attempts to visualize flagella by summing or averaging z slices, by increasing exposure time, by examining fixed specimens, and by combining amplitude and phase information (as amplitude^*^phase^2^) were all unsuccessful.

#### Bacillus subtilis

**Figure 5:**
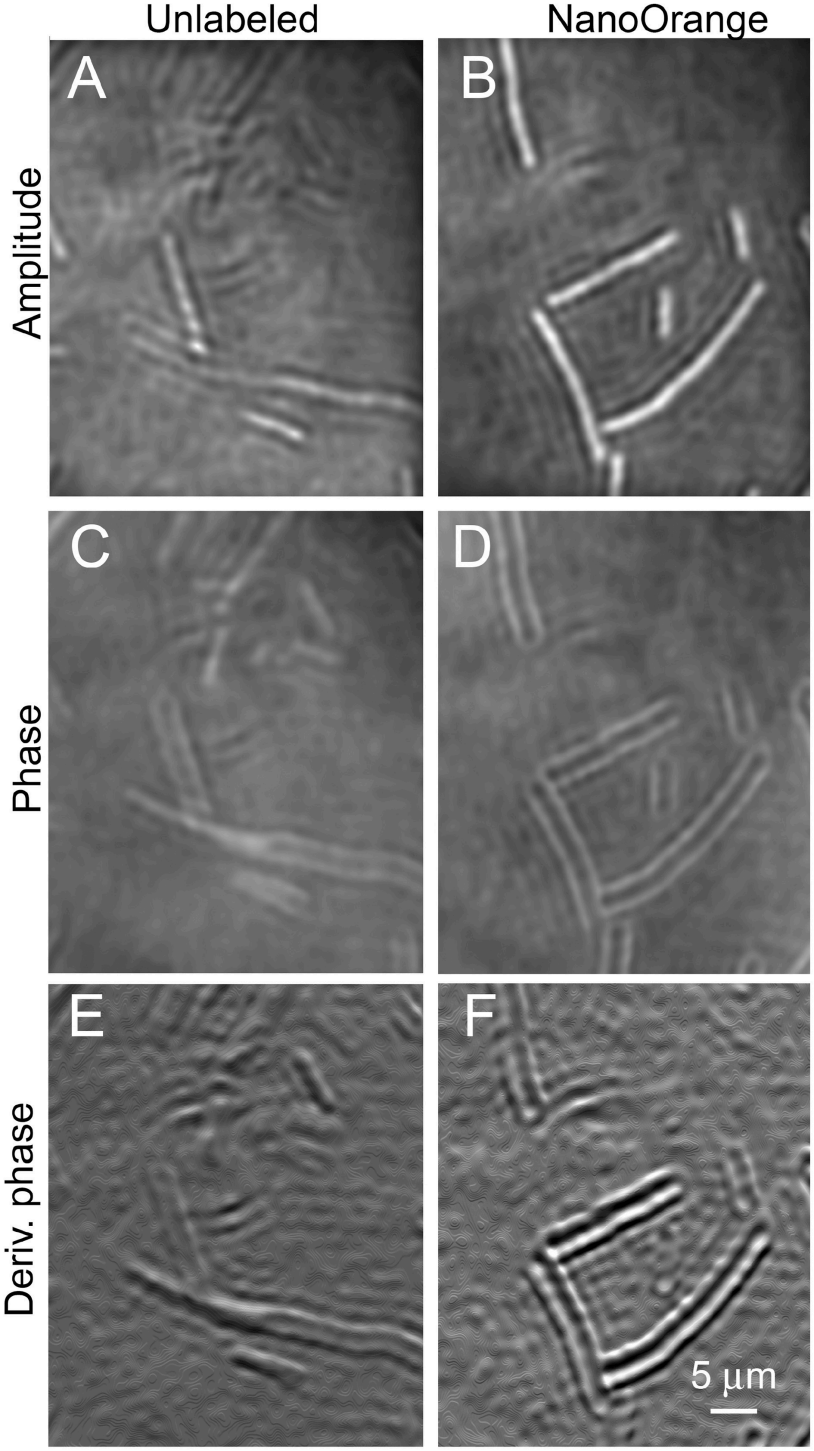
*****Bacillus subtilis*** cells unlabeled (on the left) or incubated with NanoOrange (on the right)**. Scale bar applies to all panels. Phase range is –pi to pi. **(A,B)** Amplitude images. **(C,D)** Phase images. **(E,F)** First derivative of phase in x direction.

The largest of the prokaryotic test strains, *B. subtilis*, was readily resolved in both labeled and unlabeled cultures in both amplitude and phase (Figure 5). This organism was thicker than the depth of field of the objective lens (~1 μm), so optimal images were obtained by applying a narrow filter to the reconstructions in Fourier space. This is the numerical equivalent of using an iris diaphragm to adjust the numerical aperture in light microscopy; some sacrifice of resolution is accepted for an increased depth of field, and this may be selected numerically after data acquisition. The addition of the dye clearly increased contrast around the perimeter of the cell in both amplitude (Figures 5A,B) and phase (Figures 5C,D). There was no clear subcellular structure in the amplitude or phase images. Taking the first x or y derivative of the phase, which yields an image similar to differential interference contrast (DIC), also showed enhanced edges in the labeled cells (Figures 5E,F).

We had previously observed that this concentration of NanoOrange was non-toxic to *E. coli*. Unfortunately, it caused significant toxicity to *B. subtilis*. Most of the labeled cells seen in Figure 5 are greatly elongated, indicating interference with cell division. Cell motility was nearly absent in labeled cultures (see Video 5 for normal motility in an unlabeled culture, and contrast with Video 6 showing lack of motility in a dyed culture).

**Figure 6:**
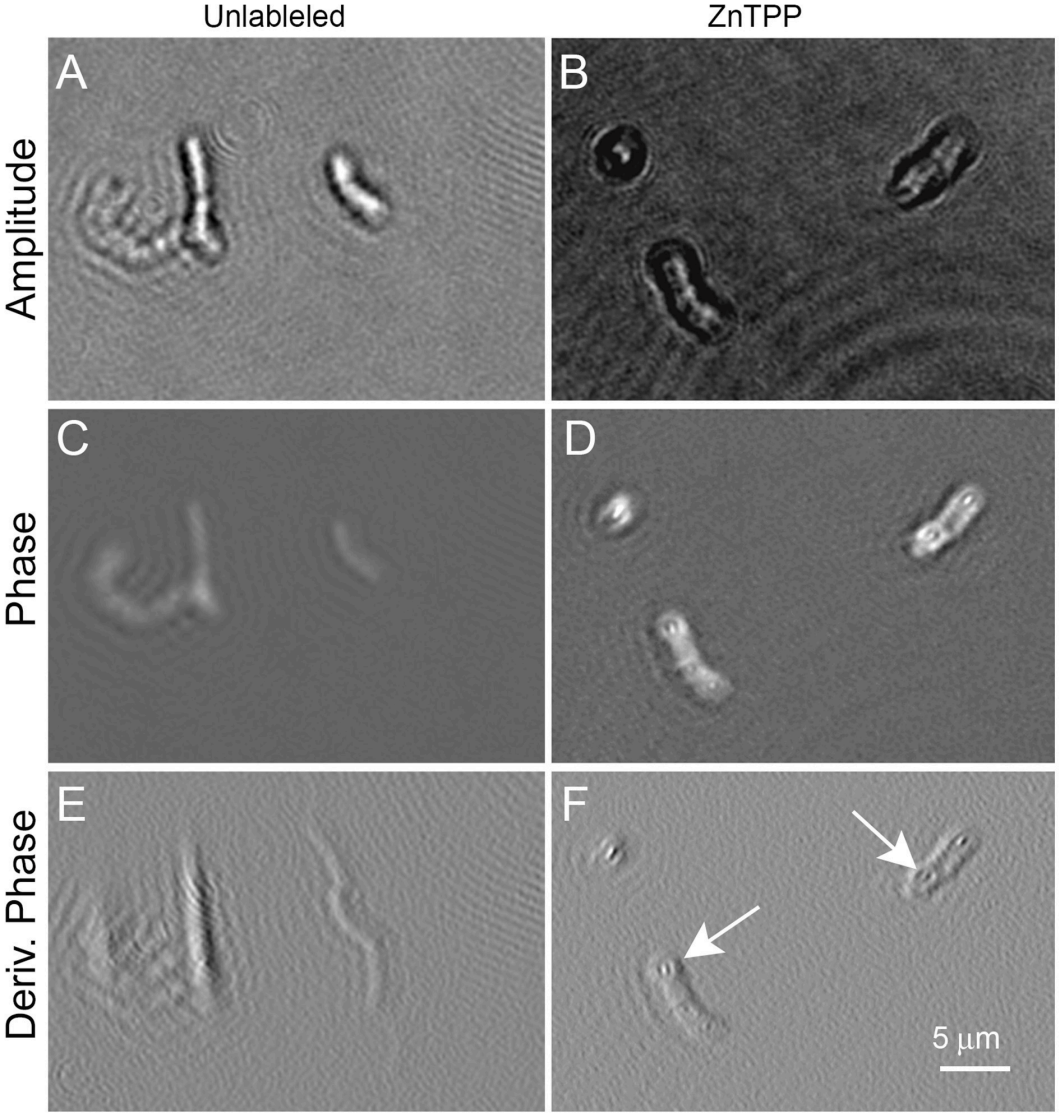
*****Bacillus subtilis*** cells unlabeled (on the left) or incubated with ZnTPP (on the right)**. Scale bar applies to all panels. Phase range is –pi to pi. **(A,B)** Amplitude images. **(C,D)** Phase images. **(E,F)** First derivative of phase in x direction. The arrows indicate areas where intracellular features may be seen. The exact identity of the features is uncertain, but their position suggests nucleoids.

Figure 6 shows *B. subtilis* labeled with ZnTPP. Again some increased contrast in both amplitude and phase was seen, particularly in the derivative of phase, where intracellular features are clearly defined. The dye was of low toxicity and the labeled cells showed normal motility (Videos 7, 8 show ZnTPP-labeled cells in amplitude and phase, respectively).

#### Escherichia coli

**Figure 7:**
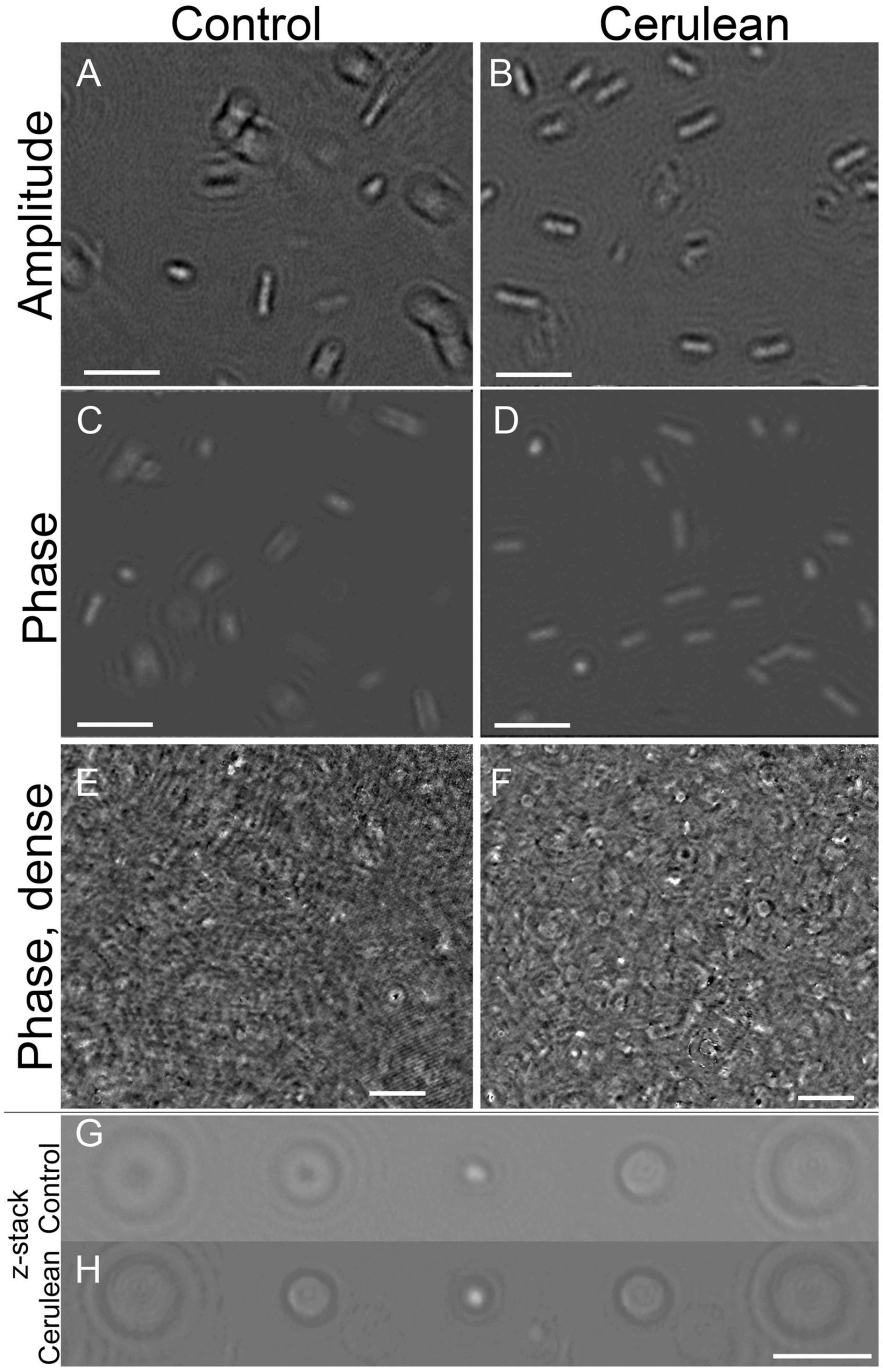
**Control cultures of ***E. coli*** (left) vs. Cerulean-expressing ***E. coli*** (right)**. Scale bar = 5 μm for all panels. Phase range is –pi to pi. **(A,B)** Amplitude. **(C,D)** Phase. **(E,F)** Dense culture, phase. **(G)** Control phase, 5 consecutive z planes of a cell positioned end-on. **(H)** Cerulean phase, 5 consecutive z planes of a cell positioned end-on.

The appearance of a control strain of *E. coli* vs. one transfected with the Cerulean fluorescent protein was examined under high resolution DHM in amplitude and phase at both 405 and 488 nm illumination. There was no substantial difference between labeled and unlabeled cells (Figures 7A–D) and no perceptible difference using the two different wavelengths (not shown). To determine whether the fluorescent protein might improve contrast in very crowded samples, cultures were prepared at the limit at which cells could be resolved without labeling (~10^8^/mL) and imaged in a 0.8 mm deep chamber. A phase image is shown in Figure 7E. Slight improvement in resolution could be seen in the Cerulean culture at the same density (Figure 7F); this was seen almost exclusively as cells viewed end-on (see also Video 9 for a phase video of an unlabeled culture and Video 10 for a phase video of a Cerulean-expressing culture, both taken at 488 nm). Examination of depth stacks (Figures 7G,H) showed a slight improvement in visibility with depth for cells positioned end-on.

#### Picocystis salinarum

**Figure 8:**
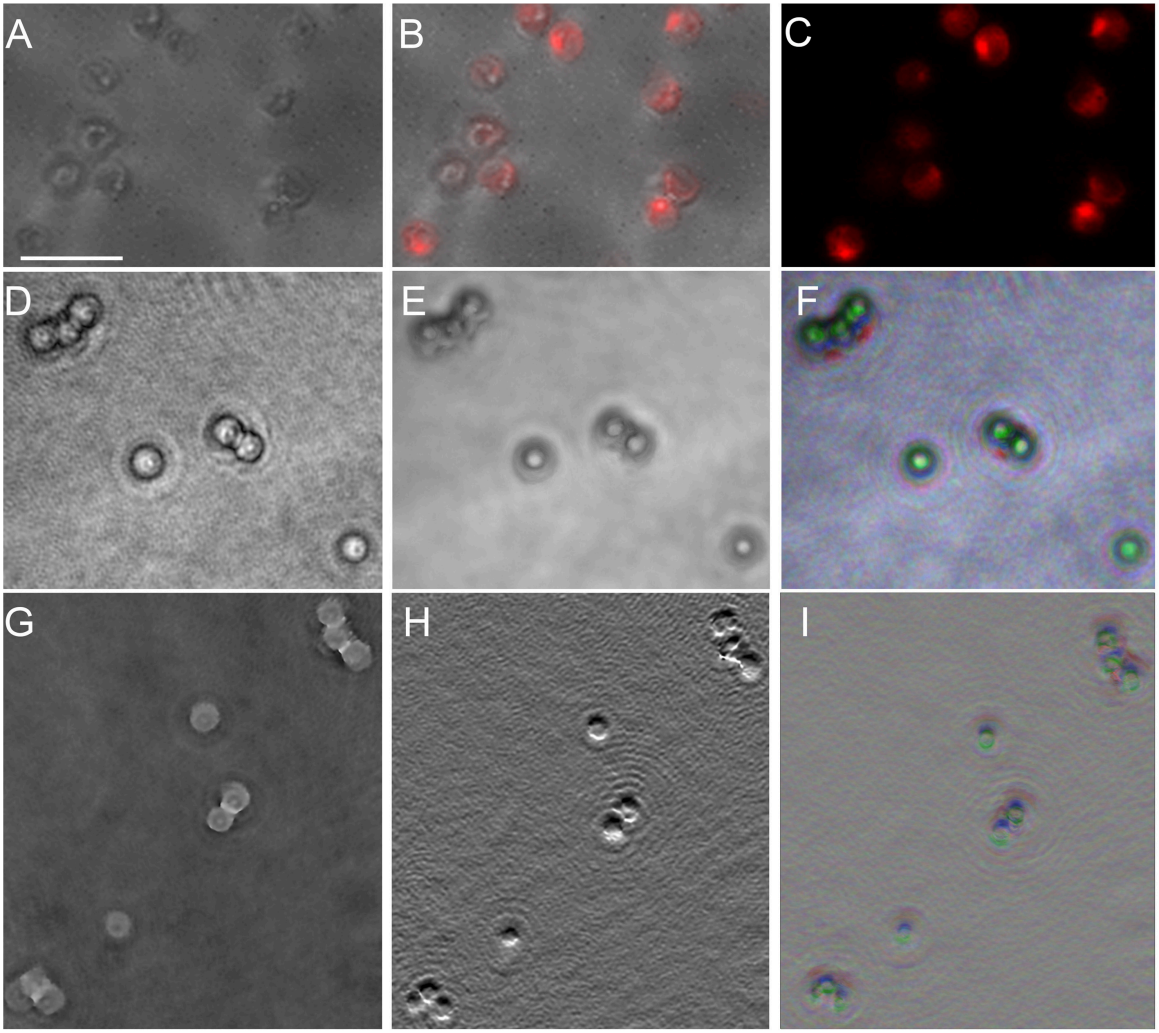
*****Picocystis salinarum*** under light microscopy and DHM**. Scale bar = 5 μm. **(A)** DIC image. **(B)** Overlay of DIC and chlorophyll fluorescence (excite 488/emit 600 LP). **(C)** Chlorophyll fluorescence. **(D)** DHM intensity image in a selected z plane. **(E)** Median z-projection of amplitude images through 150 z-planes. **(F)** Depth-coded median z-projection of amplitude images through 150 z-planes. **(G)** DHM phase image in a selected z plane. **(H)** First y-derivative of phase image in a selected plane (similar to DIC). **(I)** Depth-coded median projection of first derivative of phase through 150 z planes.

*P. salinarum* is a picoplanktonic green alga with a round cell of ~2 μm diameter composed mostly of two chloroplasts. Our samples were non-motile. Because we have previously observed significant amplitude contrast caused by chlorophyll in DHM, we examined this organism in amplitude and phase using both 488 and 405 nm illumination. The DHM images are shown compared with traditional light microscopy in Figure 8. Those taken at 488 nm are presented; there was no significant difference using 405 nm illumination (not shown). A DIC image (Figure 8A) shows the structure of the cell, with the chloroplasts demonstrating intense fluorescence (Figures 8B,C). Under 488 nm-wavelength DHM, the amplitude image clearly showed the cell outline with absorptive features corresponding to the chloroplasts (Figure 8D). A z-projection of the amplitude image showed the chloroplast structure (Figures 8E,F). The phase image (Figure 8G) and derivative of phase (Figure 8H) primarily showed the cell nucleus. A z-projection of the phase is shown in Figure 8I.

## Discussion

Imaging bacteria with light microscopy is challenging due to the small size of the cells, lack of contrast in amplitude and phase, lack of notable intracellular organelles, and rapid motility leading to loss of focus. For over a century, specific dyes and stains have been developed and discovered for improving specific contrast in brightfield and fluorescence microscopy. The use of fluorescence permits easy localization of objects well below the diffraction limit because of its ability to magnify a specific signal many-fold relative to the background. This makes fluorescence microscopy the usual tool of choice for bacterial identification and enumeration, especially in complex media such as soils, sediments, or clinical specimens.

Unfortunately, fluorescence microscopes are heavy, fragile, and demand high-power light sources and complex sample preparation, and so are ill suited to fieldwork. As a result, few bacterial imaging experiments are performed in remote locations, leaving some basic questions unanswered about many bacterial strains. In particular, are they motile, do they gain and lose motility over their life cycle, and do they exhibit taxis are questions that often cannot be answered when samples are returned to the laboratory and analyzed days to weeks after collection.

DHM provides a promising alternative to traditional widefield and fluorescence microscopy for field applications, as its optics are simple and robust and it does not require manual focusing. Many manipulations that are ordinarily performed with optical components—such as focusing and iris adjustment—may be performed digitally after the fact. The use of DHM for imaging protozoa *in situ* is well established. However, bacteria are at least 10 times smaller in radius and exhibit fewer cellular features that allow their identification, and so only a small number of studies using DHM for bacterial tracking have appeared (Molaei and Sheng, 2014; Cheong et al., 2015). Significant data processing is required to distinguish cells from optical noise inherent in the system, such as laser speckle noise, close to the diffraction limit. So far, no studies have used DHM to examine bacterial cell structure.

In this study we attempt to push the limits of DHM for imaging and tracking bacterial cells by using high-NA objectives, optimizing data analysis, and with the use of dyes to increase contrast in amplitude and/or phase. We have previously demonstrated the use of a dye with a strong blue Soret absorption band as a phase contrast enhancer. In this paper, we use a dye with a redder absorption peak (NanoOrange) to enhance primarily amplitude using a wavelength of illumination that is near the peak of the dye. This dye worked well to increase the contrast of the cell edges in both *V. alginolyticus* and *B. subtilis*. Some phase contrast enhancement was also seen. In fluorescence images, the flagella of *V. alginolyticus* could be observed, but flagella were not seen with DHM. We have previously imaged flagella of a single-celled eukaryote (*Euglena gracilis*) (Kühn et al., 2014). However, eukaryotic flagella are 11-stranded and >0.2 μm in diameter, whereas prokaryotic flagella are single-stranded and < 0.1 μm in diameter. The small size and low contrast provided by these structures did not permit their visualization here. While NanoOrange did not affect the motility of *V. alginolyticus*, it appeared to be toxic to *B. subtilis*, leading to elongated cells (indicative of DNA damage) and lack of motility. Toxicity of dyes to bacteria is strain-dependent in ways that are difficult to predict, though Gram positive bacteria are in general more sensitive to some classes of dyes, such as anilines (Kligler, 1918). Because NanoOrange has been developed primarily as a protein label (Jones et al., 2003), its toxicity to bacterial strains has not been fully explored, and further studies are needed if this stain is to be used. Other dyes with similar absorption peaks may be used to avoid toxicity. While primarily of interest for laboratory experiments, dyes may also be used in field experiments when small sample volumes are collected and studied (Nadeau et al., 2008).

We previously used a metallo-corrole dye to increase phase contrast. A similar class of dyes is the porphyrins, which show a very strong Soret band at ~400 nm. Porphyrins are widely used in biology as dyes and photosensitizers. Here we used ZnTPP to increase the phase contrast of *B. subtilis*. Internal cell structure, comparable to what is seen under light microscopy, may be observed in phase images and in the first derivative of the phase, which emulates DIC imaging.

The use of a genetically expressed cyan fluorescent protein (Cerulean) did not significantly increase the contrast of *E. coli* in either amplitude or phase, at either 405 or 488 nm illumination. This was somewhat surprising, since the extinction coefficients of the green fluorescent protein family are high (5.5 × 10^4^/M cm at peak) (McRae et al., 2005). The concentration of proteins within protein-expressing *E. coli* strains, as the current one is, has been estimated to be ~400 μM (Gather and Yun, 2011), which is at least ten-fold more concentrated than the dye as added to the cell growth medium. However, it is likely that the dye concentrates in specific subcellular regions, creating high effective local concentrations. It is possible that fluorescent proteins that create insoluble inclusions, or proteins localized to the cell membrane, would increase contrast more effectively than cytoplasmically expressed GFP. However, such inclusions may affect cell health and motility. Further development of genetically encoded dyes for DHM is clearly needed and would be of tremendous value to the field, enabling the use of DHM for many areas of cell and developmental biology.

The presence of chlorophyll leads to substantial amplitude contrast improvement under both 405 and 488 nm illumination. Using the current instrument, we were able to obtain images of a 2 μm diameter halophilic picoplankton that are comparable to or better than images taken with DIC and fluorescence microscopy. The chloroplast structure is readily observed from the increased contrast provided in amplitude by the highly absorbing chlorophyll (extinction coefficient >10^5^/M cm at 427 nm) (Strain et al., 1963). The chloroplasts do not appear in phase; the cell nucleus is the most prominent structure in the phase images. No significant differences were seen between the images taken at 405 and those taken at 488 nm.

## Conclusion

Operating Mach-Zehnder optical configurations equipped with high-NA microscope objectives at blue-wavelength regime, spatial resolution several times smaller than a bacterial cell may be achieved with DHM. DHM is highly useful as a tool for imaging three-dimensional bacterial motility, and development of contrast techniques would make it even more useful. The use of dyes to increase amplitude and/or quantitative phase contrast is still in its infancy. Very high extinction coefficients (>10^5^ M^−1^ cm^−1^ at the wavelength of illumination) and/or large concentrations of a pigment in a cell are necessary to obtain any contrast change. Dyes may assist in three-dimensional tracking, especially in crowded chambers, but their use as single-cell labeling tools is limited at this moment for DHM. Further development of data processing and labeling techniques are needed in order to localize subcellular features in bacteria such as flagella and to create genetically encoded probes that increase DHM contrast.

## Author contributions

JN: Took and analyzed holographic microscopy data. Wrote the paper. YC: Grew bacterial strains. Developed dye-labeling protocols and prepared dyed bacteria for imaging. Took fluorescence images. JK: Assisted with building the instrument. Assisted with data analysis. KL: Built and refined the instrument. Wrote the instrument description. Determined instrument performance parameters.

### Conflict of interest statement

The authors declare that the research was conducted in the absence of any commercial or financial relationships that could be construed as a potential conflict of interest.
